# *Comment to:* The Wave Sign Correlates with the Posterior Horn Medial Meniscus (PHMM) Tear in the Anterior Cruciate Ligament (ACL) Deficient Knee

**DOI:** 10.5704/MOJ.2311.018

**Published:** 2023-11

**Authors:** J Murgier

**Affiliations:** Department of Orthopaedics, Clinique Aguilera, Biarritz, France

Dear editor,

I am writing in regards to the retrospective “Wave Sign” study by Gan *et al*^1^. The authors propose that this sign correlates with the Posterior Horn Medial Meniscus (PHMM) Tear in the Anterior Cruciate Ligament (ACL) Deficient Knee. Despite a small sample size, they recommend the use of the sign as a valuable arthroscopic identification aid. In terms of the referencing, they make no reference to a strikingly similar study from 2022 by Murgier *et al*^2,3^ in which they describe an identical sign but named differently, as the crevice sign. The latter study confirmed the hypothesis that there would be a strong correlation between the presence of an unstable medial meniscal tear in patients with the crevice sign in ACL-deficient knees.

Furthermore, this was demonstrated with high specificity and positive predictive value. On the one hand, it is reassuring to find others have identified the importance of the crevice sign.

However, the lack of a valid literature search on the subject may explain the absence of appropriate referencing to a sign that has already been identified and named in the literature.
