# Supplementary material for: Comment to: The Wave Sign Correlates with the Posterior Horn Medial Meniscus (PHMM) Tear in the Anterior Cruciate Ligament (ACL) Deficient Knee
Source: Malays Orthop J. 2023 Nov;17(3):101–2. doi: 10.5704/MOJ.2311.018 (PMC10722996; doi:10.5704/MOJ.2311.018)
Supplement: Related texts

# Author’s Reply to the Letter to the Editor

**Published:** 2023-11

**Authors:** Gerrard Gan

**Affiliations:** Corresponding author, Department of Orthopaedics, Khoo Tech Puat Hospital, Singapore

Thank you for your comments. A literature search was performed prior to the commencement of the study, and the paper that first described this similar arthroscopic sign was by Zhang *et al*, 2016^1^. From there, our paper adopted their terminology in the description of this sign as the wave sign. Reviewing the commenter’s papers that were published subsequently, I realise that the pre-dated paper was not in their list of references, and may not have been identified during our literature search. Thank you again for your comments.

## References

[1] Zhang X, You T, Jiang X, Zhang H, Zhang W (2016). Characteristic arthroscopic signs of cartilage injuries indicating concomitant occult medial meniscal peripheral tears of posterior horn.. Int Orthop..
